# Morphological changes of the apical foramen after enlargement: A scoping review of *in vitro* studies

**DOI:** 10.4317/jced.61195

**Published:** 2024-05-01

**Authors:** Wesley-Viana de Sousa, Marina-da Cunha Isaltino, Luiza-de Almeida-Souto Montenegro, Bárbara-Araújo da Silva, Silmara-de Andrade Silva, Christianne Velozo, Diana-Santana de Albuquerque

**Affiliations:** 1 DDS, MSc. Department of Restorative Dentistry and Endodontics, School of Dentistry of Pernambuco, University of Pernambuco, Recife, Brazil; 2 DDS. Department of Restorative Dentistry and Endodontics, School of Dentistry of Pernambuco, University of Pernambuco, Recife, Brazil; 3 DDS, MSc, PhD. Department of Restorative Dentistry and Endodontics, School of Dentistry of Pernambuco, University of Pernambuco, Recife, Brazil

## Abstract

**Background:**

To outline the current evidence on root morphological changes after enlarging the apical foramen with NiTi instruments.

**Material and Methods:**

A search was performed in the Virtual Health Library, PubMed, EMBASE, Scopus, Web of Science, Science Direct and SciELO databases, in addition to a manual search in Google Scholar, between January 2017 and October 2023. Articles published in English that describe *in vitro* studies investigating root morphological changes after instrumentation 1 mm beyond the major apical foramen were included. The quality of evidence in the included studies was also analyzed.

**Results:**

The search retrieved 367 articles. Of these, four studies were eligible for data synthesis and analysis, all of them in vitro studies. Synthesis of the results of these *in vitro* studies showed a larger number of root morphological changes such as experimental dentinal microcraks in samples submitted to instrumentation beyond the apical foramen when compared to micro-CT images obtained before preparation.

**Conclusions:**

The *in vitro* studies analyzed in this scoping review indicate that instrumentation beyond the major foramen of the root canal, promotes morphological changes in this area and that the adoption of standardized methodologies would not only increase the accurate detection and characterization of these changes but also facilitate the application of these findings in clinical trials and patient care.

** Key words:**Endodontics, apical morphology, root canal preparation.

## Introduction

Pulp necrosis and apical periodontitis are conditions that affect periapical tissues and the entire root canal system (1,2). Studies have shown that bacteria can form a biofilm that extends into the extraradical area through the apical foramen and then remains adhered to the cementum over the root apex (3,4). Hence, effective cleaning of the root canal system requires additional care.

The intentional enlargement of the apical foramen consists of passing the instrument beyond the apical foramen during root canal treatment. This over instrumentation is commonly performed 1 mm beyond the foramen and is widely applied to teeth with periapical lesions since, in these cases, mechanical chemical preparation must be intensified due to the high bacterial load (3-5).

Foraminal enlargement can influence pain levels due to the mechanical irritation of periradicular tissues caused by rupture of the apical constriction (8). This enlargement also contributes to the extrusion of debris, which can cause periapical inflammation. The incidence of such inflammation was reported by 1.4% to 16% of patients who participated in randomized clinical trials (6), while the prevalence of pain ranged from 3% to 58% (7).

Some randomized clinical trials (9,10) have examined the impact of foraminal enlargement on postoperative pain. Silva *et al*. (9) assessed postoperative pain after enlargement of the apical foramen with manual files and concluded that both foraminal enlargement and nonforaminal enlargement caused the same level of postoperative pain. In contrast, Saini *et al*. (10), in their randomized clinical trial, concluded that foraminal enlargement with manual files increased postoperative pain intensity.

In recent years, nickel-titanium (NiTi) instruments have become essential for root canal treatment. These instruments are safer and have shown better performance than the stainless steel ones, increasing the efficiency of root canal preparation, with consequently higher success rates of root canal treatment (11). However, there is no consensus in the literature regarding the real effect of instrumentation beyond the apex, on foraminal morphology, as well as on formation of dentinal microcracks, since the published studies use different methods for the evaluation of dentinal microcracks and foraminal changes and the results are divergent (9-11).

In view of these considerations and the fact that the number of studies that address this topic is limited, this scoping review aims to map and synthesize the results of *in vitro* studies investigating apical morphological changes after enlargement of the major apical foramen with NiTi instruments.

## Material and Methods

This scoping review was structured based on the five-step framework proposed by Arksey and O’Malley (12): identification of research question; identification of relevant studies; selection of studies; data mapping; and grouping, summarizing and highlighting the results. The research question was: “Does foraminal enlargement with NiTi instruments alter apical morphology?” The JBI Manual for Evidence Synthesis (13) and the Preferred Reporting Items for Systematic Reviews and Meta-Analyses Extension for Scoping Reviews (PRISMA-ScR) checklist (14) were used. The methods of this review were registered in the Open Science Framework (https://osf.io/2xsm7/).

-Eligibility criteria

The population, concept, and context (PCC) framework was used for this study (12): population – specimens of human teeth; concept – morphological changes of the major foramen; concept – enlargement 1 mm beyond the major foramen. The eligibility criteria were *in vitro* studies published in English between January 2017 and October 2023 that evaluated the influence of apical enlargement beyond the major foramen on the formation of laboratory microcracks in the root dentin and on foraminal morphology.

Additionally, the selection of studies published from 2017 onwards aimed to incorporate the most recent advancements in the field, while ensuring methodological rigor and relevance to the research question. The exclusion criteria were carefully defined to encompass only *in vitro* studies specifically investigating apical enlargement beyond the major foramen with NiTi instruments, thereby minimizing ambiguity and ensuring alignment with the study objectives. Furthermore, duplicate articles, those not available in full-text, those not relevant to the topic, or those where instrumentation did not extend beyond the major foramen were not included in this research.

-Data sources

An active electronic search was performed independently by two groups of researchers (group 1: W.V.D.S., M.D.C.I. and B.A.D.S.; group 2: C.V., L.A.S.M. and S.A.S.) in the Virtual Health Library, PubMed, EMBASE, Scopus, Web of Science, Science Direct and SciELO, in addition to a manual search in Google Scholar, between January 2017 and January 2023.

The references selected by this search strategy were exported from the database to the Rayyan QCRI online platform (RRID:SCR_017584) in CIW/RIS format for removal of duplicate studies (15).

-Search strategy

According to the PRISMA Statement (14), the studies were selected by four reviewers (W.V.D.S., M.D.C.I., C.V., B.A.D.S.) who independently screened the titles and abstracts of all references identified by applying the eligibility criteria. Agreement between reviewers was estimated by calculating kappa scores (16). Disagreements were resolved by discussion with the final reviewer (D.S.D.A.).

For the search strategy, keywords were combined with Boolean operators as follows: 1# (“Apical enlargement’ OR “Foraminal augmentation” OR “Foraminal expansion’ OR “Foraminal debridement”), #2 (“Endodontics” OR “Root canal Instrumentation” OR “Root canal preparation” OR “Root canal therapy” OR “Root canal treatment”), and #3 (“Micro-ct” OR “Computerized micro”). No filters were applied and the search strategy was not restricted to the publication period using #1 AND #2 AND #3 ( Table 1).

-Critical assessment of evidence

The quality of the scientific evidence of the studies included in the final sample was analyzed based on the criteria of the Agency for Healthcare Research and Quality (AHRQ). The AHRQ classification is divided into six domains: I - evidence from meta-analysis of multiple randomized controlled trials (the most robust type of evidence); II – evidence from individual experimental studies; III – evidence from quasi-experimental studies; IV – evidence from descriptive studies or studies using a qualitative approach; V – evidence from case or experience studies, and VI – evidence based on expert opinion (17).

-Synthesis of evidence

The following variables were extracted from the *in vitro* studies: main author, journal, year of publication, study design, experimental methodology, and conclusion (13). Each group (group 1: W.V.D.S., M.D.C.I. and B.A.D.S.; group 2: C.V., L.A.S.M. and S.A.S.) collected and reviewed the data and the results were discussed in a consensus meeting with the coordinator (D.S.D.A.). The extracted data were then mapped for structuring this scoping review.

## Results

**Figure 1:**
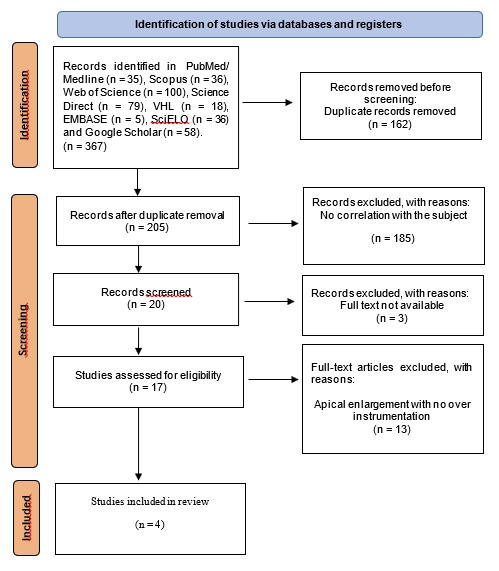
Flow diagram of the article selection process according to the Preferred Reporting Items for Systematic Reviews and Meta-Analyses Extension for Scoping Reviews (PRISMA-ScR) checklist.

The electronic search retrieved 367 articles from different databases and journals (Fig. 1). After removing duplicate articles using the Rayyan (https://www.rayyan.ai/) tool and carefully analyzing the remaining articles, only four studies (18-21) were included in this scoping review. All of them were *in vitro* studies investigating foraminal widening 1 mm beyond the greater foramen. The remaining 13 excluded articles did not perform foraminal enlargement 1 mm beyond the foramen, which is why they were not considered eligible for this scoping review.

After analysis of the relevant information of each study, a flow diagram and a Table were elaborated in order to facilitate a quick understanding of the *in vitro* studies that reported the results of foraminal enlargement 1 mm beyond the major foramen.  Table 2 shows the synthesis of the included studies: author, year of publication, sample, instruments, methodology, and conclusion. Agreement between reviewers in the study selection was as follows: PubMed/MEDLINE (kappa = 0.86), Scopus (kappa = 0.8), and remaining databases (kappa = 0.87), indicating “almost perfect agreement” between reviewers.

The studies included in this scoping review that analyzed the technique of foraminal enlargement focused more on the development of dentinal microcracks in the apical third of the root. Furthermore, these studies used NiTi instruments of different kinematics in order to determine which instrument causes more laboratory microcracks and greater morphometric changes in the major foramen (18-21).

Aggarwal *et al*. (18) analyzed the impact of foraminal enlargement on the occurrence of dentinal defects at three working lengths by micro-CT: -1 mm of the apical foramen, at the apical foramen, and +1 mm of the apical foramen. A total of 180 mesial roots of mandibular molars were instrumented using ProTaper Universal, ProTaper Gold, Twisted File Adaptive, and Reciproc Blue. Rotary instruments induced a larger number of microcracks compared to reciprocating and adaptive instruments. Instrumentation at different working lengths did not significantly influence the formation of dentinal microcracks (18).

Belladona *et al*. (19) also applied NiTi instruments at different working lengths to evaluate the formation of dentinal microcracks. Two-rooted maxillary premolars were prepared using the Reciproc R25 system (VDW, Munich, Germany). The use of that reciprocating M-Wire instrument with a high taper (25./08) short of the apical foramen, at the apical foramen, or beyond the apical foramen during the root canal preparation did not cause dentinal microcracks (19).

Using the same kinematics, Vieira *et al*. (20) analyzed not only the formation of dentinal microcracks but also morphological changes in the major foramen after the instrumentation with Reciproc Blue R25 system (VDW, Munich, Germany) at two working lengths (at and 1 mm beyond the foramen) in straight mandibular incisors and in slightly curved mesiobuccal roots of mandibular molars. Micro-CT analysis led to the conclusion that canal curvature does not influence the formation of new dentinal microcracks, nor does it significantly alter the morphometry of the major apical foramen, regardless of working length (20) since all dentinal defects identified by the analysis of postoperative scans were already present in the corresponding preoperative images.

Santos *et al*. (21) evaluated the morphometric deformation of the foramen after foraminal enlargement in 60 mesiobuccal roots of maxillary and mandibular molars that were prepared with ProTaper Universal (Dentsply Maillefer) and reciprocating Wave One (Dentsply Maillefer) instruments. The authors observed that instrumentation of the root canal at the apex or 1 mm beyond the apex promoted deformation of the major foramen, regardless of kinematics.

## Discussion

The *in vitro* studies reviewed in this scoping review indicate that root canal instrumentation leads to significant morphological changes, including foraminal enlargement and standardizing methodologies in future research, particularly in image acquisition for accurate characterization, would facilitate the translation of findings into clinical practice (19-22).

The methodology followed the five-step framework proposed by Arksey and O’Malley (12), ensuring a robust structure for the scoping review. Eligibility criteria were defined based on the PCC framework, ensuring the selection of relevant studies. Critical analysis of study quality and synthesis of results provided a comprehensive evaluation of the available evidence. However, it is important to acknowledge that inherent limitations in study selection may exist, which should be considered when interpreting findings and planning future investigations in this area.

In the realm of clinical endodontics, the determination of the apical limit for root canal instrumentation remains a subject of considerable debate. According to clinical studies, the working length should be limited to the dentinal canal, i.e., keeping the chemical-mechanical preparation within the limit of 0.5–1 mm short of the root apex (22). Nevertheless, the concept of foraminal enlargement has been proposed to improve disinfection within the apical third, leading to more favorable and predicTable treatment outcomes (18).

It’s important to note that even with the use of NiTi instruments, some changes in root morphology are is expected post-instrumentation. These changes can manifest as microcracks and alterations in foramen morphometry (23). Furthermore, evidence from *in vitro* studies indicates that extending root canal shaping 1 mm beyond the apical foramen may induce dentinal microcracks in the apical portion of the root canal. Moreover, these enlarged foramens may transport debris, irrigation solutions, and filling materials into the periapical tissues, potentially causing trauma that could disrupt tissue repair processes in both vital and non-vital teeth (24,25). These occurrences can not only exacerbate periapical inflammation but also hinder the healing process, leading to potential complications in endodontic treatment outcomes.

Previous studies have discussed the influence of mechanized instruments kinematics on the formation of these dentin defects, although the results were contrasting and inconclusive (18-20). According to Gonzalez-Sanchez *et al*. (25), an *in vitro* study showed that instrumentation beyond the canal limit with ProTaper Universal (Dentsply Maillefer) instruments, especially in curved canals, promotes the formation of an oval shape of the major foramen, thereby emphasizing the importance of understanding foraminal anatomy, since that such deformations can impact the apical sealing of the root canal obturation (25).

Moreover, recent application of artificial intelligence (AI) has yielded significant breakthroughs in anatomical comprehension, specifically concerning cone-beam computed tomography (CBCT) segmentation and three-dimensional (3D) visualization. A prior evaluation has already scrutinized the diagnostic performance of an AI system based on the deep Convolutional Neural Network (CNN) methodology for the identification of periapical pathologies within CBCT images (31-32).

Research conducted by Orhan *et al*. (33) demonstrated that an AI system successfully detected 142 out of 153 periapical lesions, achieving a reliability rate of 92.8%. These volumetric measurements obtained through deep Convolutional Neural Network (deep-CNN) analysis were comparable to those achieved through manual segmentation, demonstrating the significant potential of AI systems based on deep learning for the clinical detection of periapical pathologies in CBCT images.

When it comes to the effects of different instrumentation systems on root microcracks, the ProTaper Universal System (Dentsply Sirona, Ballaigues, Switzerland) has been associated with higher frequencies of post-instrumentation microcracks, primarily due to the design features, progressive taper, high stiffness, and continuous rotary kinematics of the instruments. On the other hand, reciprocating instrumentation tends to cause fewer microcracks than instruments with continuous rotary instruments (21).

The Reciproc Blue System (VDW, Munich, Germany) did not cause new microcracks when the working length was maintained at the apical foramen. New microcracks were observed in only a small percentage (0.67%) of post-instrumentation cases, which was not significant (20). In consonance, Liu *et al*. (26) observed microcracks in 5% of teeth prepared with Reciproc M-Wire R25 system (VDW, Munich, Germany), while Bürklein *et al*. (27) reported cracks in 60% of roots of postoperative specimens using the same instrument.

Currently, micro-CT technology is considered the gold standard to assess the formation of dentin microcracks after root canal preparation (23). This technology provides high-resolution three-dimensional images, allowing an accurate assessment of microcracks presence before and after root canal preparation in which each sample serves as its own control (24). The same does not apply to the morphological analysis of the foramen by scanning electron microscopy since the sample requires metal coating. In most cases, gold is used to reduce image artifacts and to improve visibility at higher magnifications (20).

A study comparing different methods for analyzing microcracks after root canal instrumentation has introduced the term “experimental dentinal microcracks” for changes observed in extracted teeth, as these alterations are challenging to be visualized in teeth still in the oral cavity (24). Furthermore, the advent of AI algorithms offers unprecedented analytical capabilities, with the potential to enhance clinical trials or even *in vitro* studies for assessing microcrack presence and foramen morphometric changes before and after root canal preparation through the comparison of Micro-CT’s images. However, successful utilization of AI in this context necessitates extensive training with a substantial dataset (34-35).

The limitations of this scoping review include the small number of studies that evaluated apical morphological changes after foraminal enlargement 1 mm beyond the major foramen, the heterogeneity approaches in obtaining comparative images, and the variable assessment of the results. On a positive note, certain methodological strengths in the reviewed studies, such as randomization of samples and the use of NiTi instruments with various kinematics, offer valuable reference points for future clinical trials.

## Conclusions

The *in vitro* studies analyzed in this scoping review indicate that instrumentation not only enlarges the root canal’s foramen but also induces significant morphological changes in this area. Additionally, implementing standardized methods in future studies, such as standardizing the parameters for obtaining comparative images to determine the statistical significance of the observed morphological changes, would not only increase the accurate detection and characterization of these changes but also facilitate the application of these findings in clinical trials and patient care.

## Figures and Tables

**Table 1:** Search strategy in the database.

Database	Search strategy
PubMed/ MEDLINE	("Apical enlargement" OR "Foraminal augmentation" OR "Foraminal expansion" OR "Foraminal debridement") AND ("Endodontics" OR "Root canal instrumentation" OR "Root canal preparation" OR "Root canal therapy" OR "Root canal treatment") AND ("Micro-ct" OR "Computerized micro")
Embase	apical enlargement" OR foraminal augmentation OR foraminal expansion OR foraminal debridement AND micro-ct OR computerized micro AND ((endodontics'/exp OR endodontics) AND root canal instrumentation OR root canal preparation OR root canal therapy OR root canal treatment)
SciELO	("Apical enlargement" OR "Foraminal augmentation" OR "Foraminal expansion" OR "Foraminal debridement") AND ("Micro-ct" OR "Computerized micro") AND ("Endodontics" OR "Root canal instrumentation" OR "Root canal preparation" OR "Root canal therapy" OR "Root canal treatment")
Web of Science	((ALL=(("Apical enlargement" OR "Foraminal augmentation" OR "Foraminal expansion" OR "Foraminal debridement"))) AND ALL=(("Micro-ct" OR "Computerized micro"))) AND ALL=(("Endodontics" OR "Root canal instrumentation" OR "Root canal preparation" OR "Root canal therapy" OR "Root canal treatment"))
Scopus	(( "apical enlargement" OR "Foraminal augmentation" OR "Foraminal expansion" OR "Foraminal debridement" ) ) AND ( ( "micro-ct" OR "computerized micro" ) ) AND (( endodontics" OR "root canal instrumentation" OR "root canal preparation" OR "root canal therapy" OR "root canal treatment" ) )
Virtual Health Library	("Apical enlargement" OR "Foraminal augmentation" OR "Foraminal expansion" OR "Foraminal debridement") AND ("Endodontics" OR "Root canal instrumentation" OR "Root canal preparation" OR "Root canal therapy" OR "Root canal treatment) AND ("Micro-ct" OR "Computerized micro")
Science Direct	("Apical enlargement" OR "Foraminal augmentation" OR "Foraminal expansion" OR "Foraminal debridement") AND ("Micro-ct" OR "Computerized micro") AND ("Endodontics" OR "Root canal instrumentation" OR "Root canal preparation" OR "Root canal therapy" OR "Root canal treatment")
Google Scholar	("Apical enlargement" OR "Foraminal augmentation" OR "Foraminal expansion" OR "Foraminal debridement") AND ("Micro-ct" OR "Computerized micro") AND ("Endodontics" OR "Root canal instrumentation" OR "Root canal preparation" OR "Root canal therapy" OR "Root canal treatment")

**Table 2:** Synthesis of the in vitro studies that investigated foraminal enlargement 1 mm beyond the major foramen.

Authors	Year of publication	Journal	Sample	Methodology	Conclusion
Aggarwal et al. (18)	2021	Journal of Endodontics	Mesial roots of mandibular molars (n=180)	Preparation: 1 mm short of at and 1 mm beyond the major apical foramen. Micro-CT before and after root canal preparation with ProTaper Universal SX-F2, ProTaper Gold SX-F2, TF Adaptive SM1-SM2, and Reciproc Blue R25.	Rotary instrumentation induced a larger number of dentinal microcracks compared to reciprocating and adaptive instruments. Instrumentation at different working lengths did not significantly influence dentinal microcrack formation.
Belladonna et al. (19)	2021	International Endodontics Journal	Maxillary premolars (n=20)	Preparation: 1 mm short of, at and 1 mm beyond the major apical foramen. Micro-CT scanning was performed after each instrumentation with Reciproc R25.	Reciprocating root canal preparation short of, at or beyond the apical foramen did not cause dentinal microcracks.
Vieira et al. (20)	2020	Journal of Endodontics	Mandibular incisors (n=30) and mandibular first molars (n=30)	Two subgroups: SEM and micro-CT. Roots in the SEM group were instrumented at the limit of the apical foramen and 1 mm beyond the foramen, and those in the micro-CT group were instrumented 1 mm short of, at the limit, and 1 mm beyond the foramen. All roots were instrumented with Reciproc Blue R25.	The preparation of straight and moderately curved root canals with Reciproc Blue, regardless of working length, did not influence the occurrence of apical foramen deformations and did not cause dentinal microcracks.
Santos et al. (21)	2018	Journal of Endodontics	Mesiobuccal roots of mandibular and maxillary molars (n=60)	Both groups were instrumented with ProTaper Universal and Wave One Primary at the level of the apex and 1 mm beyond the apex using rotary kinematics in the former and reciprocating kinematics in the latter.	Root canal instrumentation at the apex or 1 mm beyond the apex promoted greater foraminal deformation, regardless of kinematics.

## Data Availability

No data were used to support the study.
